# Cinematic rendering to improve visualization of supplementary and ectopic teeth using CT datasets

**DOI:** 10.1259/dmfr.20230058

**Published:** 2023-04-04

**Authors:** Ines Willershausen, Fabian Necker, Roman Kloeckner, Corinna Lesley Seidel, Friedrich Paulsen, Lina Gölz, Michael Scholz

**Affiliations:** 1 Department of Orthodontics and Orofacial Orthopedics, Friedrich-Alexander-University Erlangen-Nürnberg, Gluecksstrasse, Erlangen, Germany; 2 Incubator for Medical Mixed Reality at Stanford (IMMERS), Department of Radiology, Stanford School of Medicine, Stanford, United States; 3 Institute of Functional and Clinical Anatomy Friedrich-Alexander-University Erlangen-Nürnberg, Krankenhausstrasse, Erlangen, Germany; 4 Institute of Interventional Radiology University Hospital of Schleswig-Holstein-Campus Lübeck, Ratzeburger Allee, Lübeck, Germany

**Keywords:** CT, CBCT, volume rendering, 3D visualization, dental radiology, dental anatomy

## Abstract

**Objectives:**

Ectopic, impacted, and supplementary teeth are the number one reason for cross-sectional imaging in pediatric dentistry. The accurate post-processing of acquired data sets is crucial to obtain precise, yet also intuitively understandable three-dimensional (3D) models, which facilitate clinical decision-making and improve treatment outcomes. Cinematic rendering (CR) is anovel visualization technique using physically based volume rendering to create photorealistic images from DICOM data. The aim of the present study was to tailor pre-existing CR reconstruction parameters for use in dental imaging with the example of the diagnostic 3D visualization of ectopic, impacted, and supplementary teeth.

**Methods:**

CR was employed for the volumetric image visualization of midface CT data sets. Predefined reconstruction parameters were specifically modified to visualize the presented dental pathologies, dentulous jaw, and isolated teeth. The 3D spatial relationship of the teeth, as well as their structural relationship with the antagonizing dentition, could immediately be investigated and highlighted by separate, interactive 3D visualization after segmentation through windowing.

**Results:**

To the best of our knowledge, CR has not been implemented for the visualization of supplementary and ectopic teeth segmented from the surrounding bone because the software has not yet provided appropriate customized reconstruction parameters for dental imaging. When employing our new, modified reconstruction parameters, its application presents a fast approach to obtain realistic visualizations of both dental and osseous structures.

**Conclusions:**

CR enables dentists and oral surgeons to gain an improved 3D understanding of anatomical structures, allowing for more intuitive treatment planning and patient communication.

## Introduction

Ever since its introduction in dentistry almost 20 years ago, cross-sectional imaging has been widely used in dental medicine.^1,2^ Two-dimensional (2D) X-rays such as panoramic and lateral cephalometric images, which are routinely performed in clinical treatment planning, show certain limitations, namely superimposition on other anatomical landmarks, imaging artifacts, distortion, and a restricted field of view.^3,4^ In particular, complex dental and skeletal conditions call for more informative cross-sectional imaging.^5^

According to the DIMITRA guidelines, impacted and supplementary teeth are considered the “prime and justified indication” for cross-sectional imaging in pediatric dentistry.^6^ There is substantial evidence that cone beam CT (CBCT) improves the quality of dental treatment and, if needed, the outcome of a respective surgical intervention.^7^ Traditionally, studies devoted to the diagnosis of impacted/supplementary teeth on cross-sectional imaging evaluated the respective teeth in the classical 2D axial, coronal, and sagittal planes.^8,9^ Because a considerable portion of spatial information is lost when only looking at the aforementioned 2D planes and superimposition on other anatomical landmarks impedes a precise diagnosis, clinicians and researchers have been striving for virtual lifelike 3D models. The manual segmentation of cross-sectional data sets has been implemented to obtain three-dimensional (3D) models; however, this technique is elaborate, time-consuming, and especially error-prone. More recently, automatic segmentation techniques have been established, promising a faster segmentation of both jaws.^10,11^ In contrast to classical segmentation techniques, volume rendering has the advantage that it is always based on unprocessed, original patient data. Therefore, non-destructive editing is possible without ever deleting, modifying, or losing original information in contrast to manual volume segmentation processes, where data subsets are created.^12^

Cinematic rendering (CR) has recently been introduced as an alternative approach for visualizing volumetric medical imaging data and advancing conventional volume-rendering algorithms.^13,14^ This technology is especially applicable for data derived from CT and cone beam CT (CBCT). Because both modalities are used for 3D diagnosis in dentistry, an application here seems particularly suitable. Originally, CR was inspired by the rendering techniques from the movie industry that sparked the creation of increasingly realistic digital 3D animation movies (influencing it being named “cinematic”).^15^ Building on top of novel physically based light-simulation algorithms, billions of virtual light rays are simulated every second in a physically accurate manner. In contrast, the more unrealistic conventional volume rendering typically only uses a single directional light source (similar to a flashlight), limiting a realistic appearance.^10,14^ Different realistic lighting environments with reflections, shadows, and highlights, as well as the light diffusion within the tissues of the human body, are major contributors in creating a photorealistic visualization. Including these lighting complexities, images produced with CR are therefore much closer to reality compared with pre-existing techniques. Since its introduction, CR has found applications in clinical research and anatomical education.^16–18^ In medical education, CR has been described as a very promising tool to alleviate comprehension of human anatomy and respective pathologies.^17^ Regarding bony anatomy, cinematic rendering improves visualizations of skull sutures, lower extremity and pelvic fractures, and skeletal dysplasia.^19–22^ To date, current CR implementations have not been appropriately customized for dental imaging and do not include adequate reconstruction parameters tailored to the specific needs of dental imaging, such as readily segmenting hard dental tissue from the surrounding bone so that the isolated dentition is clearly visible. So far, only limited evidence on the use of CR in dental imaging is available, with a strong focus on fractures and carcinoma as maxillofacial indications.^23,24^

To the best of our knowledge, our group is the first to introduce CR for the diagnostic visualization of ectopic, impacted, and supplementary teeth.

## Methods

**Figure 1.**
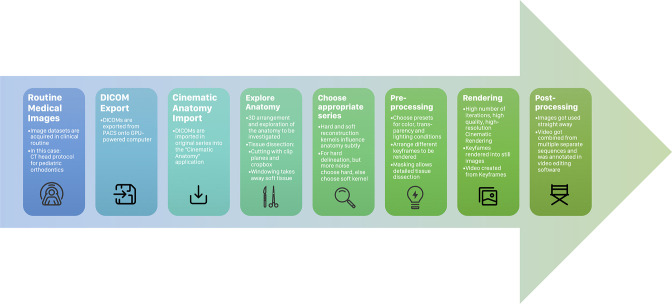
Displays the post-processing pipeline of volumetric data for 3D visualization by means of cinematic rendering. 3D, three-dimensional; DICOM, Digital Imaging and Communications in Medicine; GPU, graphics processing unit; PACS, picture archiving and communications system.

In total, three default DICOM data sets, which had been acquired in clinical routine due to the presence of ectopic, impacted, and supplementary teeth, were retrospectively selected from our archives. After the approval of the institutional review board (IRB Number: 22-126-Br), all patients and legal guardians provided written informed consent for the publication of their CT images. The Cinematic Anatomy application (Siemens Healthineers GmbH, Erlangen,Germany) was employed to visualize (DICOM) image data using CR according to a predefined post-processing pipeline (Figure 1)^18,25^ The existing reconstruction parameters for bony anatomy were optimized to highlight the different midface anatomical structures, such as the maxilla and mandible, as well as the isolated dentition. We further modified a second set of reconstruction parameters including semi-transparency and dedicated colors for the sufficient differentiation of several dental tissue types, *e.g.* the distinction between enamel, dentine, and pulp, as well as tooth morphology (tooth crown *vs* tooth neck in different colors, and bone *vs* tooth asopaque and semitransparent). Colorization within the reconstruction parameters is performed using a special transfer function that translates monochrome intensity values from the original image slices to color values on a predefined gradient including transparency values. In addition, we used soft tissue kernels (Case 1: H31s; Case 2: Hr40s; Case 3: H31s) and hard tissue kernels (Case 1: H70h; Case 2: Hr60s; Case 3: H70s) to distinguish teeth in close spatial relationships. Soft kernels are used when aiming at a naturally looking overall appearance in the context of CR. However, for the discrimination of two teeth in proximity, we used the same CT scan reconstructed with a harder kernel instead. By using arbitrarily positioned clip planes and a crop box, the data set was split into regions of interest (ROIs) depending on the respective anatomy to be highlighted in each image. Windowing was performed in analogy to windowing in typical radiology applications, where a subset of intensity values is chosen for display (*e.g.* similar to a conventional lung or bone window). As structures with higher contrast (bone, teeth) have higher Hounsfield units (HUs) compared with structures with lower contrast (soft tissue, muscles), one can intuitively select which anatomical structures to display depending on their respective intensity values. Therefore, using CR, a virtual dissection can quickly be performed by choosing a specific window that only displays structures of certain HU values, *e.g.* the jaw and teeth, without performing any additional segmentation steps. For fine-tuned segmentation, a 3D mask tool was used, which allows the user to directly interact with the 3D visualization to remove parts that should not be visualized (*e.g.* remnants of bone that were not fully removed solely by windowing), further enhancing the desired visualization outcome. All edits were performed non-destructively and can be undone at any time, allowing iterative improvements and modifications. All modifications were combined and subsequently stored in keyframes that were later exported as high-resolution JPEG images (native 4K resolution, 3840 × 2160 pixels, high-quality rendering settings). Additionally, by arranging a carefully selected subset of keyframes on a storyboard, a set of fluent animations was generated and exported as conventional MP4 movies (native HD resolution, 1920x1080 pixels, 80%quality rendering settings). The rendering process for each image took around 60 s, with the movie exporting process taking around 12–24 h (depending on the image complexity in each video sequence), which was influenced by the high number of image frames needed depending on video length. Rendering was conducted on a high-performance computer with a dedicated graphics processing unit (LENOVO Legion 7, 2021 Edition, AMD Ryzen^™^ 9 5900 HX Processor, 32 GB RAM, 2 × 1 TB SSD, GeForce RTX 3080 Mobile (with 16 GB of GPU Memory)). Separate video sequences per case were edited, cut, and combined in DaVinci Resolve (DaVinci Resolve 18, Blackmagic Design, Melbourne, Australia). During the exploration and creation process, the image can be interactively adjusted in realtime, with the image being slightly noisy at first and iteratively sharpening as the course of an increasing number of light rays through the tissue is simulated (approximately 2–4 s in this setup).

## Results

**Figure 2.**
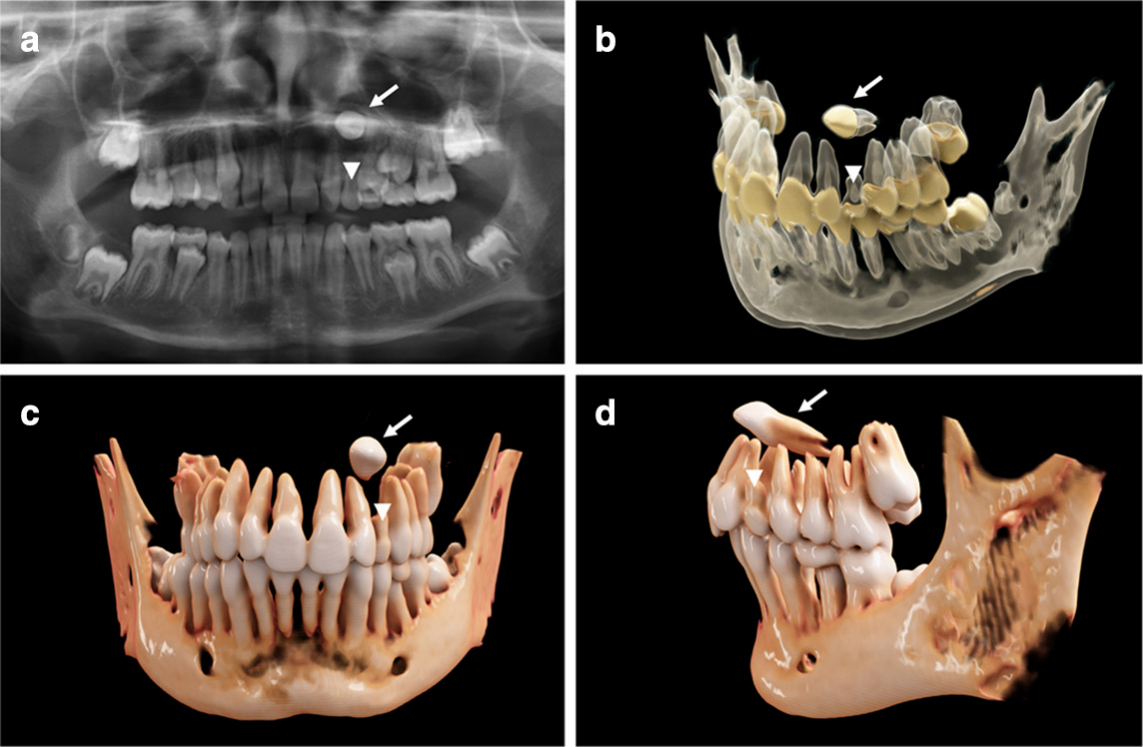
An 11.7-year-old girl with a horizontally impacted canine (white arrows, Figure 1a–d) and a persisting deciduous 63 canine (white arrowheads, Figure 1a–d). (**a**) A panoramic radiograph is displayed. (**b**) Semi-transparent reconstruction parameters are utilized to visualize both bone and teeth, as well as the different dental tissues. (**c, d**) The individual dentition and bone tissue with a soft kernel show a photorealistic visualization in frontal and lateral view.

**Figure 3.**
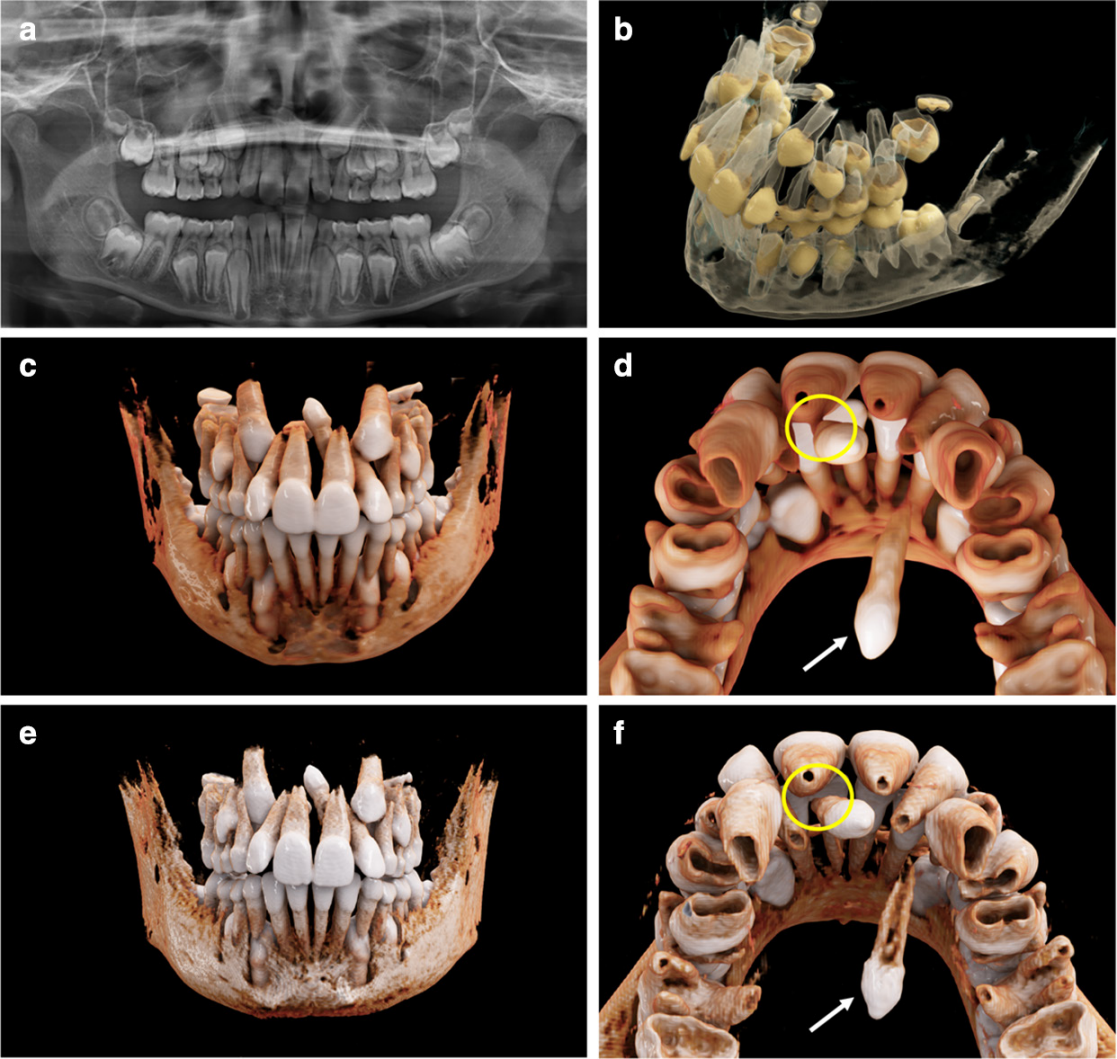
An 11.2-year-old boy with a mesiodens and a further supplementary tooth (white arrows; 3D, **f**) within the hard palate. (**a**) A panoramic radiograph is displayed. (**b**) Semi-transparent reconstruction parameters are displayed. (**c, d**) The bone reconstruction parameters with a soft kernel can lead to artifacts in regions with teeth near each other (yellow circle, in d). (**e, f**) The same situation is shown with a hard kernel, allowing a better differentiation between the roots of the mesiodens and of tooth 21 (yellow circle in f).

**Figure 4.**
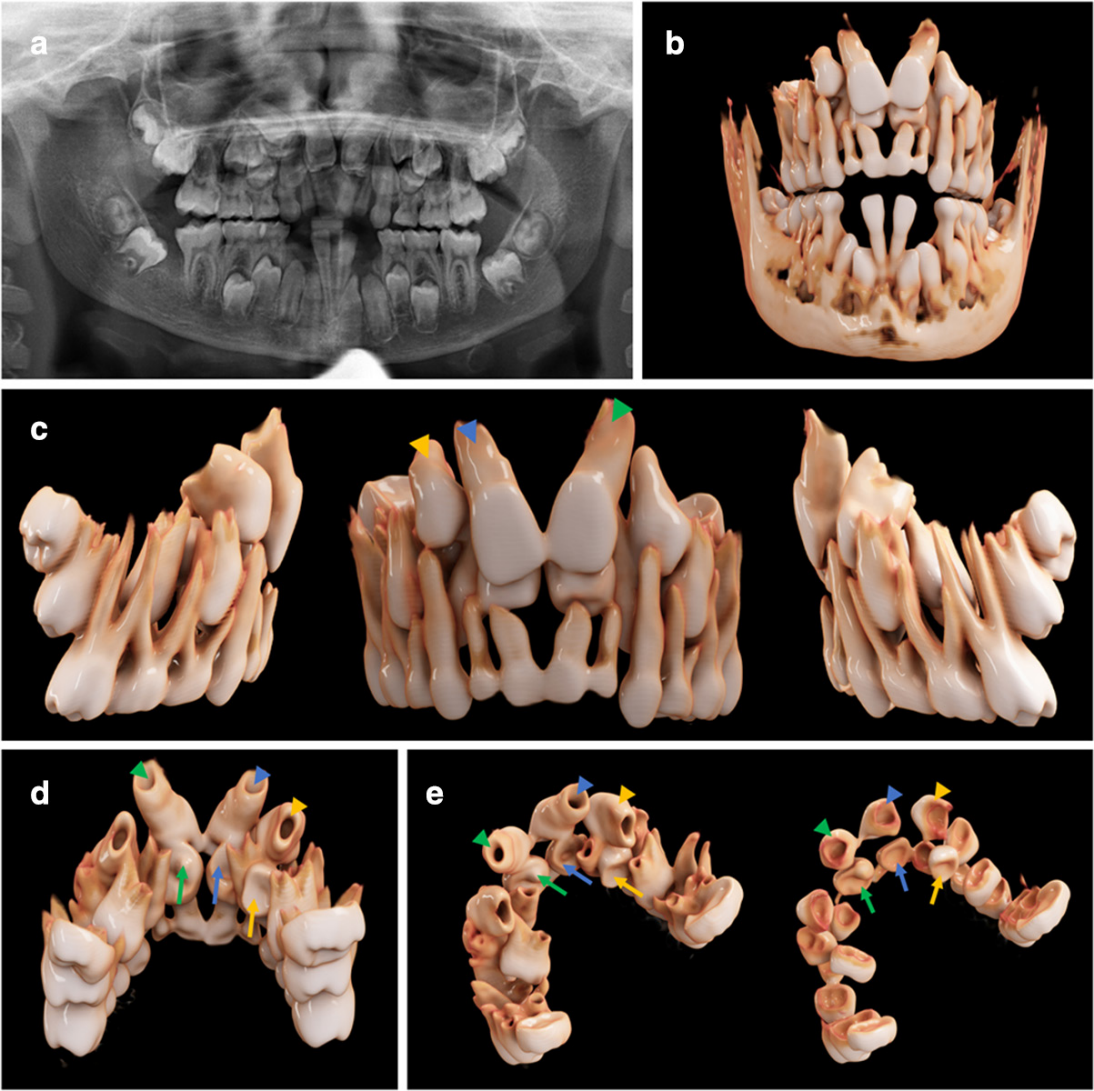
A 12.4-year-old boy with cleidocranial dysostosis and multiple supernumerary and impacted teeth. (**a**) The panoramic image is displayed. (**b-e**) The bone present with a soft kernel in the frontal, lateral, and palatal view is illustrated. (**c-e**) Tooth 11 (blue arrowhead), tooth 21 (green arrowhead), and tooth 13 (yellow arrowhead) are displayed, as well as their respective supernumerary teeth 11 (blue arrow), 21 (green arrow), and 13 (yellow arrow).

CR was successfully employed to three-dimensionally visualize complex orthodontic cases with ectopic, impacted (Figures 2 and 3), and supernumerary teeth (Figures 3 and 4). When utilizing the first reconstruction parameters for bony anatomy, the teeth and bone are displayed as fully opaque with varying shades of light brown and the tooth crown going into shades of white. This yields a close resemblance with real anatomy and was used preferably when aiming at a lifelike image (Figures 2 and 3c, d; Figure 4b–e). In the second reconstruction parameters, tooth crowns are displayed yellowish with roots, with pulp camber and bone being semi-transparent and glass-like (Figures 2 and 3b). Due to the semi-transparency, both teeth and bone can be illustrated simultaneously, allowing a perspective on the teeth’s respective positioning within the bony jaw. Because different dental tissues can be distinguished by means of this second preset, it is particularly useful when intending to display the precise anatomy of the pulpal chamber, *e.g*. in cases of tooth gemination. As mentioned above, segmentation difficulty through windowing lies in the relative similarity of HU values for different tissue types (*e.g.* teeth and bone). A balanced compromise must be reached when choosing the appropriate window for the sufficient differentiation of anatomical structures. This can lead to situations where two anatomical structures (*i.e.* 2 teeth: tooth 21 and a mesiodens (Figure 3d)) blend into one another, impeding a clear differentiation of the separate entities, mainly due to the soft kernels that already caused slight blurring in the original images. We normally use soft kernels for their natural-looking overall appearance in the context of cinematic rendering (*e.g.* Figure 3c and d; Figure 4d). However, for the discrimination of two teeth in close proximity, we used the same CT scan reconstructed with a harder kernel instead (Figure 3e and f). Harder kernel reconstructions possess a higher local spatial resolution and sharper edges that enable better differentiation between adjacent structures; however, they also introduce noise, which is amplified during volume rendering, leading to visualizations, which are overall less optically appealing. Therefore, we only employed the harder reconstruction kernels in circumstances of anatomically unclear spatial relations between teeth due to blurred blending.

### CR visualization for ectopic canines

In the first case, we present an 11.7-year-old girl in the late mixed-dentition phase. Cross-sectional imaging was performed due to an ectopic canine 23, which was horizontally impacted and located in the immediate vicinity of the cavum nasi and adjacent premolars (white arrow, Figure 2a–d). The deciduous and partially resorbed tooth 63 was still *in situ* (white arrowheads, Figure 2a–d). A panoramic radiograph of the dentition is shown in Figure 2a. In Figure 2b, the semi-transparent reconstruction parameters are used to visualize both the mandibula, comprising the lower dentition, and the teeth of the upper jaw, which have been fully segmented from the maxillary bone. Semi-transparency is helpful when different dental tissues are to be distinguished, illustrated by the example of the tooth germ 38 (Figure 2b), which is still impacted in the lower jaw and not detectable when using soft kernel reconstruction (Figure 2c and d). In Figure 2c and d, bone reconstruction parameters with a soft kernel (H31s; 1 mm slice thickness) were used to create a photorealistic, true-to-real anatomy image of the dentition. Comparable to Figure 2b, the lower incisors have been partially segmented from the mandibula to illustrate this feature in the bone reconstruction parameters. A 3D review of this case, which incorporates the reconstruction parameters for bony anatomy with a soft kernel, is shown in Supplementary Video 1.

Supplementary Video 1.

### CR visualization for mesiodens and supplementary teeth

Next, we present the case of an 11.2-year-old boy with a family history of hypodontia. Figure 3a displays a panoramic radiograph of the individual dentition. In Figure 3b, the semi-transparent reconstruction parameters display a 3D overview of the intraoral situation. In addition to a mesiodens (visible in Figure 3b–f), which was already diagnosed on the panoramic image, a further supplementary tooth (white arrows Figure 3d and f) was confirmed in the volumetric visualization. This supplementary tooth is located within the hard palate, with a close spatial relationship to the cavum nasi. Figure 3c and d illustrate the bone reconstruction parameters with a soft kernel (Hr40s; 0,6 mm slice thickness). The mesiodens and tooth 21 seem to have an interconnection in this illustration (Figure 3d, yellow circle), which must be considered as an artifact of the standard kernel reconstruction. In Figure 3e and f, the same excerpt is displayed, reconstructed with a hard kernel, allowing a better differentiation of teeth 21 and the mesiodens. When utilizing the hard kernel (Hr60s; 0,6 mm slice thickness), the real spatial situation becomes obvious (Figure 3f; yellow circle)at the expense of losing parts of the photorealistic impression. For a detailed visualization and comparison of the soft and hard kernel, see Supplementary Video 2.

Supplementary Video 2.

### CR visualization for cleidocranial dysostosis

In the third case, a 12.4-year-old boy with cleidocranial dysostosis is presented. The patient displays three supernumerary and rudimentary teeth, namely tooth 11 (blue arrow), 21 (green arrow), and 13 (yellow arrow), which are located palatal to the regular teeth 11(blue arrowhead), 21 (green arrowhead), and 13 (yellow arrowhead) (Figure 4c–e). All permanent incisors are still impacted in the upper jaw and the respective deciduous teeth persisted. Figure 4a shows the panoramic image of the dentition. For this complex case, the standard soft kernel reconstruction (Hr31s; 0,6 mm slice thickness) (Figure 4b–e) was used to obtain a photorealistic visualization. These true-to-life images are particularly beneficial in cases with multiple supplementary teeth, where the differentiation between the different teeth in the data set is always challenging, especially when only using cross-sectional images. In Figure 4e,the palatal view of the dentition is given, with arrowheads and arrows pointing at the aforementioned incisors of the upper dentition. On the left, this figure illustrates the visualization of the entire teeth (with crowns and roots), and on the right, the spatial situation of the isolated crowns is presented, respectively (Figure 4e). The crowns have been digitally isolated from the roots to reduce blurring and thus allow a better differentiation between the single teeth. For a detailed visualization of the soft kernel and the semi-transparent reconstruction parameters, see Supplementary Video 3.

Supplementary Video 3.

## Discussion

In recent decades, cross-sectional imaging has been established as the gold-standard in the diagnosis of complex orthodontic cases. CT/CBCT provides 3D data sets, facilitating the accurate discrimination of high-density structures, such as bone and teeth.^26^ 3D imaging allows both dental treatment and, if needed, surgical intervention to be planned more precisely, improving treatment outcomes and reducing overall treatment time.^7,27^ In dental radiology, a slice-based 2D visualization (MPR planes) of teeth and the adjacent bony structures in classical axial, coronal, and sagittal planes is still predominant.^9,28^ For improved volumetric visualization, manual and, more recently, fully automated rendering techniques, such as volume rendering, have been utilized in dental medicine.^29^

Our study introduces CR as a novel rendering technique for the photorealistic volumetric visualization of dental DICOM data sets. Using the masking and windowing functionality of the Cinematic Anatomy application (Siemens Healthineers), upper and lower jaw and respective dentitions were easily segmented, creating natural-appearing images for true-to-life anatomy representations. In the diagnosis of impacted and ectopic teeth, the spatial relationship with the antagonizing dentition is considerable because root resorptions of adjacent teeth have to be ruled out on the basis of 3D analysis. Furthermore, being able to isolate the dentition from the surrounding bone is highly beneficial for a 3D understanding of the teeth’s spatial relationship, particularly in complex cases with multiple supernumerary teeth (Figure 4). Hence, the significantly improved and more precise 3D understanding derived from CR could be influential in creating correct pre-treatment evaluations and better subsequent treatment plans. Our observations are consistent with those of other studies, which describe CR as enhancing spatial depth perception as well as 3D understandings of investigated anatomical structures.^15,30^ The 3D visualization of bony anatomy has already been depicted in the literature, primarily focusing on factures, for the dentomaxillofacial region, as well as the lower extremities and pelvic ring.^19–21^ Those studies support our findings that CR improves 3D visualization while simultaneously shortening diagnosis time.

In addition, CR is helpful for patient education, enhancing their understanding of their underlying disease, resulting in an increased compliance while simultaneously strengthening the patient–physician interaction.^31^ This might be particularly appealing to pediatric dentistry, where children and parents alike may find lifelike 3D rendered images easier to understand than conventional 2D cross-sectional imaging.

However, because CR is a novel technique, it still has limitations regarding dental imaging. Until now, the software did not provide reconstruction parameters tailored specifically towards visualizing dental anatomy, necessitating our approach to adapt pre-existing reconstruction parameters designed for other anatomical structures. Due to the significant and discontinuous contrast differences (HU values) of dental and surrounding tissues in the virtual dissection process, structures such as dental apices obtain blur or have fuzzy edges. This is because balance during windowing needs to be achieved, namely balancing the removal of soft tissue while maintaining the clear edge definitions of the higher-contrast bony jaw and teeth. In the case of multiple supplementary teeth, the technique still has certain limitations, with the teeth crowns blurring into one another. This can partially be addressed by specifically using harder reconstruction kernels, as described above (Figure 3e and f). The current reconstruction time of 12–24 h is not reasonable for routine clinical application. However, we anticipate that due to software updates, the reconstruction time will soon significantly decrease. Furthermore, the reconstruction time will equally speed up when conducted with stronger computing systems, such as high-performance workstations with several dedicated GPUs.

In conclusion, this study was able to introduce CR as a promising new technique in orthodontic 3D imaging. It offers the possibility to create 3D models from volumetric data sets that can be segmented and rendered with relative ease. These images can significantly improve the assessment of anatomical structures for orthodontists and oral surgeons while simultaneously enabling more intuitive treatment planning and patient communication.
